# Identification and Functional Analysis of Rare HECTD1 Missense Variants in Human Neural Tube Defects

**DOI:** 10.21203/rs.3.rs-3794712/v1

**Published:** 2024-01-02

**Authors:** Elias Oxman, Huili Li, Hong-Yan Wang, Irene Zohn

**Affiliations:** Children’s National Hospital; University of Colorado at Boulder; Fudan University; Children’s National Hospital

**Keywords:** Neural tube defects, HECTD1, extracellular HSP90, cranial mesenchyme

## Abstract

Neural tube defects (NTDs) are severe malformations of the central nervous system that arise from failure of neural tube closure. HECTD1 is an E3 ubiquitin ligase required for cranial neural tube closure in mouse models. NTDs in the *Hectd1* mutant mouse model are due to the failure of cranial mesenchyme morphogenesis during neural fold elevation. Our earlier research has linked increased secretion of extracellular heat shock protein 90 (eHSP90) to aberrant cranial mesenchyme morphogenesis in the Hectd1 model. Furthermore, overexpression of HECTD1 suppresses stress-induced eHSP90 secretion in cell lines. In this study, we report the identification of five rare *HECTD1* missense sequence variants in NTD cases. The variants were found through targeted next-generation sequencing in a Chinese cohort of 352 NTD cases and 224 ethnically matched controls. We present data showing that *HECTD1* is a highly conserved gene, extremely intolerant to loss-of-function mutations and missense changes. To evaluate the functional consequences of NTD-associated missense variants, functional assays in HEK293T cells were performed to examine protein expression and the ability of HECTD1 sequence variants to suppress eHSP90 secretion. One NTD-associated variant (A1084T) had significantly reduced expression in HEK293T cells. All five NTD-associated variants (p.M392V, p.T801I, p.I906V, p.A1084T, and p.P1835L) reduced regulation of eHSP90 secretion by HECTD1, while a putative benign variant (p.P2474L) did not. These findings are the first association of *HECTD*1 sequence variation with human disease and suggest that sequence variation in *HECTD1* may play a role in the etiology of human NTDs.

## Introduction

Neural Tube Defects (NTDs) are severe congenital malformations of the spine and brain. They are among the most common structural birth defects, with a global prevalence of 0.5 to 10 per 1000 live births (Greene and Copp 2014). The occurrence of NTDs in various populations is affected by genetics, geography, and the maternal diet (Li et al. 2006; Liu et al. 2016). For instance, in Northern China’s Shanxi Province in 2003, the frequency of NTDs was 138.7 per 10,000 births, 10-fold higher than in the United States and Europe (Li et al. 2006; Morris and Wald 2007; Williams et al. 2015). The diet in this region was deficient in folate, and the incidence of NTDs in this region was significantly reduced following a campaign to provide folic acid supplementation (Liu et al. 2016; Meng et al. 2015). NTDs arise in the first trimester of pregnancy from defects in neurulation, a process where the neural plate transforms into a tube to form the central nervous system (Wallingford et al. 2013). NTDs can occur at different axial levels, resulting in anencephaly in the head, spina bifida in the spine, or craniorachischisis with the complete failure of neural tube closure along the entire neural axis. Anencephaly and craniorachischisis are fatal, whereas spina bifida can result in significant disability.

While the causes of NTDs are polygenic and multifactorial, estimates suggest that ~ 70% of NTDs have a significant genetic component (Copp et al. 2015; Jorde et al. 1983; Lupo et al. 2017). For instance, the relative risk among first-degree relatives increases to 3% (Jorde et al. 1983), and NTDs are more frequent in some genetic syndromes and chromosomal anomalies (Copp et al. 2015; Lupo et al. 2017; Toriello and Higgins 1983). Genome-wide sequencing projects indicate that rare and novel variants in NTD candidate genes may contribute to NTDs in an oligogenic fashion (Chen et al. 2018c; Ishida et al. 2018). For example, screening a cohort of 90 cases with cranial NTDs from northeast England between 1992 and 2011 with a targeted exome sequencing panel of 191 genes identified 397 rare variants (Ishida et al. 2018). On average, NTD cases had nine rare/novel variants, three of which were predicted to be damaging. In contrast, case-control samples had an average of two novel/rare variants, with 1.5 predicted to be damaging (Ishida et al. 2018). In analyzing whole-genome sequencing (WGS) data from three different NTD cohorts (Han Chinese, Caucasian USA, and Middle Eastern/Qatar) with various NTD types, researchers found a higher occurrence of singleton loss-of-function (SLoFVs) variants among NTD cases than controls (Chen et al. 2018c). SLoFVs were defined as variants that appear only once in the 1000 genome project. Based on these findings, the authors suggest that the number of SLoFVs is a stable and reliable genomic indicator of NTD risk in humans, with nine SLoFVs a genomic threshold for NTD risk (Chen et al. 2018c).

Animal studies have identified hundreds of genes involved in forming the neural tube (Harris and Juriloff 2007, 2010; Ishida et al. 2018; Wilde et al. 2014). Searching for rare and novel sequence variants in these NTD candidate genes in NTD cases has been a powerful tool for revealing the genetic causes of human NTDs (Ishida et al. 2018; Wolujewicz and Ross 2019). Rare variants in genes under constraint are of particular interest in rare disease research due to the anticipated stronger effects (Gudmundsson et al. 2022). However, an analysis of the Genome Aggregation Database (gnomAD v4.0.0) (Chen et al. 2022; Karczewski et al. 2020) reveals that many NTD candidate genes tolerate missense variation (Supplemental Table 1). For instance, multiple *VANGL1* missense variants have been identified in NTD cases and the pathogenesis of many of these variants was validated in experimental assays (Bartsch et al. 2012; Cai et al. 2014; Cheng et al. 2021; De Marco et al. 2014; Doudney et al. 2005; Fatima et al. 2022; Humphries et al. 2020; Iliescu et al. 2014; Iliescu et al. 2011; Kibar et al. 2009; Kibar et al. 2007; Merello et al. 2015; Reynolds et al. 2010; Tian et al. 2020a; Tian et al. 2020b; Wang et al. 2018). *VANGL1* tolerates missense but not loss of function (LOF) variants with a missense Z score of 0.59 but a loss intolerance probability (pLI) score close to 1 (0.91) and a LOF observed/expected upper bound fraction (LOEUF) score of 0.53. This is consistent with the proposed digenic and multigenic origins of NTDs involving sequence variation in *VANGL* genes (Juriloff and Harris 2012; Torban et al. 2008; Wang et al. 2018; Zohn 2012; Zohn and Sarkar 2008). In contrast, only a handful of NTD candidate genes exhibit substantial selection against missense variation. Among these is *HECTD1*, a HECT domain E3 ubiquitin ligase that targets proteins for degradation or alters their function. Since causal LOF variants for Mendelian and severe complex diseases are enriched in ‘mutation intolerant’ genes (Agarwal et al. 2023), the strong selection against LOF and missense variants in *HECTD1* provides support to the idea that deleterious sequence variation in the *HECTD1* gene would significantly affect embryonic development and based on the mouse phenotype, disrupt neural tube closure (Sarkar and Zohn 2012; Zohn et al. 2007).

We originally identified the mouse *Hectd1* gene in an ENU mutagenesis screen to identify genes required for neural tube closure (Kasarskis et al. 1998; Zohn et al. 2007). This novel ENU-induced *Hectd1* mutant mouse model (*openmind, opm*) exhibited fully penetrant exencephaly in homozygous *Hectd1*^opm/opm^ embryos and incomplete penetrance in heterozygotes (Zohn et al. 2007). Interestingly, depending on the mutation, 5–20% of heterozygous *Hectd1* mutant mouse embryos showed exencephaly (Zohn et al. 2007). Our study of the developmental mechanism leading to exencephaly in the *Hectd1* mouse model revealed that the defect arises from the abnormal morphogenesis of the cranial mesenchyme (Sarkar and Zohn 2012; Zohn et al. 2007), a process required to elevate the cranial neural folds (Morris-Wiman and Brinkley 1990a, b, c; Morriss and Solursh 1978a; Morriss and Solursh 1978b; Zohn and Sarkar 2012). Analysis of the pathways regulated by HECTD1 in the cranial mesenchyme implicated increased secretion of extracellular Hsp90 (eHsp90) as a likely cause of the cranial mesenchyme and neural tube closure defects in *Hectd1* mutant embryos (Sarkar and Zohn 2012). Our research demonstrated that eHSP90 stimulates the migration of cranial mesenchyme cells, interfering with normal cranial mesenchyme morphogenesis and neural fold elevation. Likewise, the expression of HECTD1 in HEK293T cells suppresses the stressed-induced secretion of eHSP90 (Sarkar and Zohn 2012).

The present study identifies five case-specific missense variants in the *HECTD1* gene from a Chinese NTD cohort. Based on our prior knowledge that NTDs in the *Hectd1* mutant mouse model are due to elevated secretion of eHSP90 stimulating cranial mesenchyme migration (Sarkar and Zohn 2012), we utilize this as an assay to functionally test the impact of missense variants on HECTD1 function. Our findings indicate that all variants associated with NTDs showed activity loss, whereas a putative benign *HECTD1* variant did not. These data suggest sequence variation in *HECTD1* may contribute to human NTDs.

## Materials and Methods

### Human Subjects and Targeted Next-Generation Sequencing of HECTD1

The subjects of this study were previously described (Chen et al. 2018a; Chen et al. 2018b; Lei et al. 2019; Qiao et al. 2016; Shi et al. 2018; Ye et al. 2020). 352 NTD samples were collected from aborted fetuses and children with spina bifida from the 1990s to the 2010s in five Chinese provinces. Tissue samples were collected from 309 aborted fetuses (23.4 ± 6.7 weeks) with craniorachischisis, encephalocele, anencephaly, exencephaly, spina bifida, or more than one type of NTD. Whole blood samples were obtained from 43 children (6.4 ± 4.6 years) with spina bifida. 224 control samples (58.9% female, 40.6% male, 0.5% unknown) were ethnically and gender-matched unrelated healthy volunteers recruited from Shanxi (aborted healthy fetuses) and Shanghai (blood samples from healthy first-year college students) in China. Protocols were reviewed and approved by the Ethics Committee of the School of Life Sciences, Fudan University. The coding region of *HECTD1* was sequenced by targeted next-generation sequencing as previously described (Qiao et al. 2016). Variants were assessed for predictive deleteriousness using SIFT (http://sift.jcvi.org/) and Mutation Taster2 (http://www.mutationtaster.org/). *HECTD1* variants analyzed in this study were absent in controls, the 1000 genome project (http://www.1000genomes.org), or extremely rare in the Genome Aggregation Database (gnomAD; http://gnomad.broadinstitute.org/).

### Cell Culture and DNA Transfection

HEK293T cells from American Type Culture Collection (CRL-11268) were maintained in high glucose DMEM media (Gibco 11995065) supplemented with 10% Fetal bovine serum and Penicillin-Streptomycin and grown in a 5% CO2 humidified incubator at 37°C. *pCMV-HA-Hectd1* and *pCMV-Hsp90a-Myc* constructs were previously described (Sarkar and Zohn 2012). Aligent QuikChange II XL Site-Directed Mutagenesis (Aligent 200521) was used to introduce missense mutations confirmed by Sanger sequencing. *CD63-pEGFP C2* (gift from Paul Luzio, Addgene plasmid 62964; https://www.addgene.org/62964/; RRID: Addgene_62964), *pRK5-HA-GFP* (HA-GFP was a gift from Carol Mercer (Addgene plasmid 137763; http://n2t.net/addgene:137763; RRID: Addgene_137763) (Stefely et al. 2020). Plasmids were prepared using the Qiagen Mini-Prep Kit (Qiagen 27104) and cut with PvuII-HF restriction enzyme (NEB R3151S) to confirm quality and concentration. HEK293T cells were transfected using Lipofectamine 3000 Reagent (L3000008) according to the manufacturer’s instructions with either 730 ng *pCMV-HA-Hectd1*, 400 ng *pCMV-Hsp90a-Myc*, and 50 ng *CD63-pEGFP C2* or 1 ug *pCMV-HA*-*Hectd1* and 50 ng *pRK5-HA-GFP*.

### Western Blot Analysis

Transfected cells were expanded to a 6-well plate 24 hours after transfection and lysed 48 hours post-transfection. Lysis was performed using IP Lysis Buffer (Pierce, 87787) with diluted 100x Halt Protease Inhibitor Cocktail (78429). Plates were rocked at 4°C for 10 minutes, scraped, and debris was separated by centrifuging 13,000 × g for 10 minutes at 4°C. The supernatant was collected, and concentration was quantified using a Pierce BCA Protein assay kit (23225) or Coomassie Protein Assay Reagent (Thermo Scientific 1856209). Lysate was prepared in NuPAGE 4x Sample Buffer (NP0007), NuPAGE Reducing Agent 10x (NP0004), heated at 70°C for 10 min, and resolved on 10-well NuPAGE 3 to 8%, Tris-acetate Mini Protein Gel (EA0375) with NuPAGE Tris-Acetate SDS Running Buffer (LA0041) to detect HA-HECTD1 or 10-well NuPAGE 4 to 12%, Bis-Tris Mini Protein Gel (NP0321) with the NuPAGE MES SDS Running Buffer (NP0002) to detect HA-GFP run at 150V for 1.5 hours. Protein was transferred using a low fluorescence PVDF Transfer Membrane (22860) at 20V for 1.5 hours. Transfer buffer was supplemented with NuPAGE Transfer Buffer 20x (NP0006), 10% Methanol, and NuPAGE Antioxidant (NP0005). Membranes were blocked with LiCOR Intercept Blocking Buffer (927–60,001) for 1 hour at room temperature. Primary antibodies were incubated overnight at 4°C at the following dilutions: HA-HECTD1 and HA-GFP were detected using anti-HA.11 (Clone 16B12, BioLegend) diluted 1:750 and 1:1000, respectively, in Intercept Antibody Dilutant (927–65,001). Secondary antibody IRDye 800CW donkey anti-mouse (92,632,212; 1:15,000 dilution) was incubated for 1 hour at room temperature. Blots were imaged using the LiCor Odyssey CLX imaging system and LI-COR software. Exported image files were processed to 800 channel only, inverted LUT, and integrated density was measured over 3 established Regions of Interest (ROIs). Relative expression was measured through averaged integrated density values and calculated by normalizing HA-HECTD1 expression to transfection control HA-GFP. Statistical analysis was performed using Prism GraphPad 9, and the significance was determined using the two-tailed Student’s t-test. Four replicates were used in the western analysis; p.A1084T is reported in triplicate due to a technical error. HA-HECTD1 and p.P2474L were assayed using the same method independently for three replicates.

### eHsp90a Secretion Assay

Transfected cells were passaged 24 hours after initial transfection to a 6-well plate with a sterilized coverslip in the well. 24 hours after passaging cells, respective wells were treated with 10 micromolar N-acetyl- N-Acetyl-Leu-Leu-Nle-CHO (ALLN, BML-P120) for 1 hour. Cells were washed with 1x PBS with calcium and magnesium (Gibco 14040141) and fixed for 20 minutes with 4% Paraformaldehyde. Blocking buffer for primary antibodies and washes were formulated with 1x PBS (Sigma, P3813) and 1% heat-inactivated goat serum (16210064). Primary incubations were done for 1 hour at room temperature with Myc-Tag (71D10) Rabbit mAb (1:200 Cell Signal, 2278), and secondary incubations for 1 hour at room temperature in the dark with Hoechst 33342 Solution (1:500, 62249) and Alexa-Fluor Anti-Rabbit 555 (1:250, A-21428). Final washes were performed with a blocking buffer with 0.1% Triton - X100 to reduce excess signal. Coverslips were mounted using Fluoromount-G^™^ Mounting Medium (00–4958-02). Slides were imaged using the Leica TCS8 Confocal Microscope with a 63x-magnification oil immersion objective (NA = 1.4). Image acquisition was made blind by randomization of slides. Images were acquired on detection of CD63-GFP transfection control, and then parameters were adjusted for detection of extracellular Hsp90a or lack thereof. The maximum projection of the Z stack was applied to images in ImageJ. Images of cells acquired during blind acquisition had to exhibit standard nuclei shape and health to ensure intact plasma membrane. Final images were processed using a coding key and scored by five independent reviewers to reach a consensus on categorizing myc-eHSP90 staining as “No secretion,” “Moderate secretion,” or “Excessive secretion.” After the scoring was performed, slides were unblinded for statistical analysis. Chi-squared tests were performed to test significance. ALLN treatment conditions were assessed independently. Three replicates were performed, consisting of five frames per replicate for 15 frames per condition. Hectd1 and p.C2579G were n = 20 after including p.P2474L in a separate assay.

### Multiple Sequence Alignment of HECTD1 Protein Sequences

To obtain the sequences for the graphical alignment and multiple sequence alignment of HECTD1, a series of blastp queries were performed strategically, moving through lower-order organisms. The subject sequence was obtained from UniProt as the canonical HECTD1 *Homo sapiens* protein sequence (Q9ULT8). Standard laboratory animal models were first queried (*M. musculus, G. gallus, X. laevis, D. rerio, D. melanogaster, C. elegans*). Additional queries were performed: opossums (taxid: 9265), bony fish (taxid: 7898), lampreys (taxid: 7745), cnidaria (taxid: 6073), sponges (taxid: 6040), and anasdipea (taxid: 6497). Named proteins and X1 isoforms (where applicable) were chosen as sequences for multiple sequence alignment. blastp (https://blast.ncbi.nlm.nih.gov/Blast.cgi?PAGE=Proteins) queries were performed using default parameters. Sequences were aligned using MUSCLE with the UPGMA clustering method, 1.20 hydrophobicity multiplier, −2.90 gap open, and Lambda of 24. Alignment was visualized utilizing SnapGene.

### AlphaFold Rendering of Human NTD-Associated Variants

AlphaFold v2.3.0 (Jumper et al., 2021) renderings were performed on the Children’s National Research Institute High-Performance Computing cluster. The human canonical sequence (Uniprot Q9ULT8) was edited into multiple FASTA files with missense variants. Each missense variant FASTA protein sequence was run using the monomer_ptm preset and 9/8/2022 max template date. The zero-ranked PDB output for each variant was imported into YASARA and superimposed on the zero-ranked wild type PDB output. Residues of interest were transformed to the ball and stick view for structural and confidence score (b-factor/pLDDT) analysis. pLDDT scores are included for wild type and missense residues.

## Results

**Figure 1:**
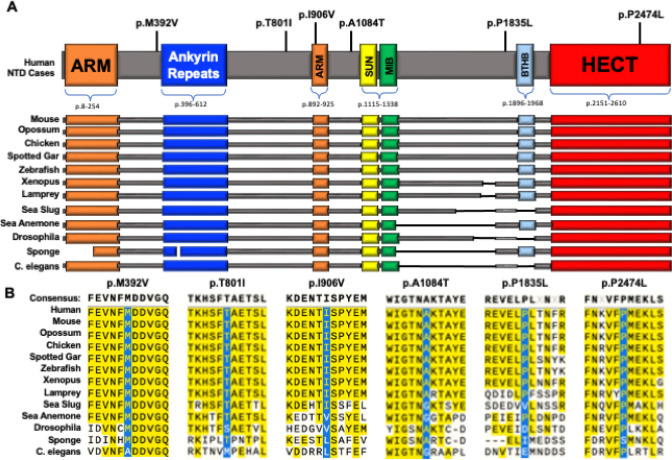
NTD-Associated Missense Variants in *HECTD1*. A. Domain structure of HECTD1 showing armadillo (ARM, orange) and ankyrin repeats (ANK, blue), Sad1/UNC (SUN, yellow), mind-bomb domain (MIB, green), Basic Tilted Helix Bundle (BTHB, light blue), and Homologous to the E6-AP Carboxyl Terminus domain (HECT, red) domains. The position of human NTD-associated missense variants and the putative benign p.P2474L variant are indicated. B. The multiple-sequence protein alignment was done using MUSCLE. The amino acid sequences adjacent to the NTD-associated missense variants are shown in the various HECTD1 orthologues. Sequences were obtained through Blastp. The accession numbers, % query coverage, and E-value are listed in Table 2. The amino acids altered by missense variants are absolutely conserved among vertebrates, with some conservation into invertebrate taxonomic groups

Targeted next-generation sequencing of *HECTD1* in an NTD cohort identified five rare (minor allele frequency < 0.01) heterozygous missense variants (Table 1 and Fig. 1A). Evaluation of these variants across publicly available databases indicated that variants are either exceedingly rare or unique. The c. 1174A > G (p. M392V), c. 2716A > G (p. I906V), c. 2716A > G (p. A1084T) and c.5504C > T (p.P1835L) were rare, with gnomAD frequencies of 2.09e-6 (2.723e-6 in the European non-Finnish but not reported in the East Asian ancestry group), 1.86e-5 (2.233e-5 among the East Asian ancestry group), 7.97e-6 (1.389e-4 among the East Asian ancestry group), and 6.84e-7 (8.996e-7 in the European non-Finnish but not reported in the East Asian ancestry group), respectively. One variant, c.2402C > T (p.T801I), was novel and not found in the gnomAD dataset or among the ethnically matched control cohort. All but one variant (p. I906V) is predicted to be damaging/deleterious by PolyPhen-2 and SIFT variant effect prediction tools (Adzhubei et al. 2010; Ng and Henikoff 2003). As a control, this study also includes a reported (ClinVar) benign variant, p.P2474L (rs111683057). This variant has a gnomAD frequency of 1.1e-3, primarily in the African/African American ancestry group (2.043 e-2), with 9 homozygous individuals reported.

The *HECTD1* missense variants alter conserved amino acids localized within defined and well-conserved functional domains of HECTD1 or amino acids just flanking these domains (Fig. 1A). Our previous results indicate that HECTD1 interacts with HSP90 through N-terminal armadillo and ankyrin repeats (Sarkar and Zohn 2012). SUN (Sad1-UNC-84 homology) domain and Basic tilted helix bundle (BTHB) domains can also mediate protein-protein interactions (Dilworth et al. 2017; Starr and Fridolfsson 2010). A multiple sequence alignment was performed (Fig. 1B) utilizing MUSCLE (MUltiple Sequence Comparison by Log-Expectation) to align the sequences of selected species (Starr and Fridolfsson 2010). All residues implicated in our missense variants were universally conserved among vertebrates, and several were also preserved in the invertebrate species surveyed.

**Figure 2:**
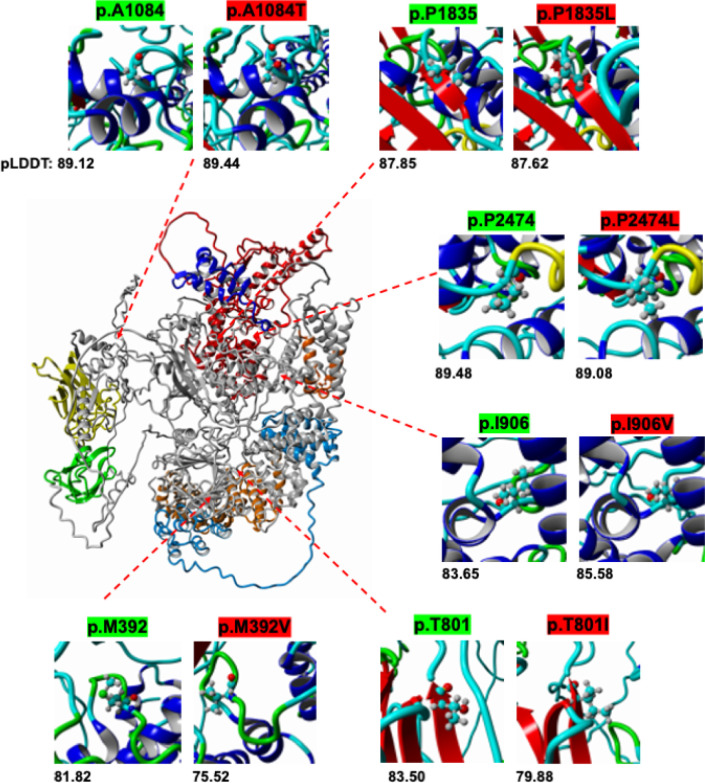
AlphaFold Rendering of HECTD1. Rendering displays a color overlay of defined domains matching the color scheme shown in Fig. 1A. Residues of interest are magnified and illustrated in ball & stick format. The coloration of magnified images depicts the pLDDT scores of respective residues. Models are shown superimposed and aligned based on the highest-ranked output

AlphaFold analysis of HECTD1 protein structure suggests missense variants do not result in significant changes to tertiary or secondary structure (Fig. 2). The pLDDT scores do not indicate relevant deviations from the wild type structure. Differences in pLDDT scores are represented in coloration changes of the b-factor field in the 3D rendering of the PDB output and text output shown underneath each frame (Fig. 2). Significant conformational changes would have been indicative of destabilizing variants.

### HECTD1 is a Highly Conserved and Constrained Gene

Sequence variation in human *HECTD1* is under notable constraint. The gnomAD database indicates that loss of function variants are significantly underrepresented in the *HECTD1* gene. *HECTD1* loss of function variants have a LoF intolerant (pLI) score of 1 and a LOEUF score of 0.27 (50 observed vs. the expected 238.4 LOF variants). Since haploinsufficient genes often have a high pLI score and a low LOEUF score (Gudmundsson et al. 2022; Lek et al. 2016), these data indicate that *HECTD1* could be a haploinsufficient disease gene. The *HECTD1* gene is also highly constrained for missense variation with a Z score of 6.42 (1911 observed SNVs vs. 2849.1 expected SNVs). Moreover, *HECTD1* has an RVIS (Residual Variation Intolerance Score) of −2.23, ranking among the 1.33% most intolerant to functional genomic changes of all human genes (Petrovski et al. 2013). These metrics suggest that missense variation and loss of function changes in *HECTD1* are poorly tolerated compared to most human genes. Additionally, this high level of constraint could be consistent with mutations in *HECTD1* causing disease in heterozygous individuals.

### The p.A1084T Variant Showed Reduced Expression in HEK293T Cells

**Figure 3:**
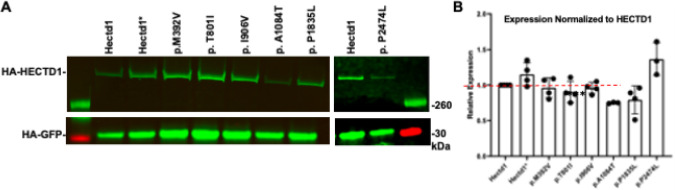
Expression of *HECTD1* Missense Variants in HEK293T Cells. A. Western blot analysis of *HA-Hectd1* expression levels in HEK293T cells co-transfected with *HA-GFP* as a transfection control. B. Expression was normalized to HA-GFP and then wild type HECTD1 and averaged across four independent experiments. Transfection of the NTD-associated variant, p.A1084T, showed a significantly reduced expression level (p < 1e-6, n=3) compared to the wild type. Differences in expression of the other NTD-associated variants (n=4), putative benign p.P2474L (n=3) and p.C2579G (Hectd1*) were not significant (p>0.05)

First, we tested if missense variants alter the expression levels of HECTD1 in HEK293T cells. Western blot analysis of transiently transfected HEK293T cells shows that some *HECTD1* variants from human NTD cases reduced expression compared to the wild type HECTD1 (Fig. 3). The p.A1084T variant exhibited significantly reduced expression in three independent experiments (*p* < 1e-6), whereas the expression of the other NTD-associated variants and the benign p.P2474L variant was not significantly reduced.

### HECTD1 NTD-Associated Variants Show Reduced Activity

**Figure 4:**
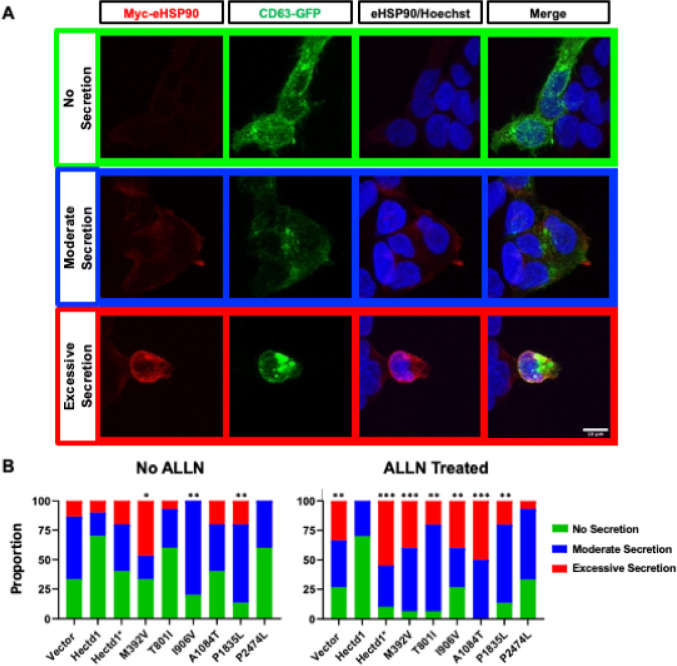
Functional Analysis of *HECTD1* Missense Variants in HEK293T cells. A. Representative images of activity scored for eHSP90 secretion: No secretion (Green), moderate secretion (Blue), and excessive secretion (Red). Co-transfected CD63-GFP is shown to identify transfected cells. Blocking and incubation of primary antibodies were performed without detergent to ensure the detection of extracellular eHSP90. **B.** Relative distributions of activity score per frame is shown as a percentage of the total number of frames scored. Three replicates were performed, consisting of 5 frames per replicate for a total of 15 frames per condition. Replicates were scored by each of 5 observers to reach a consensus on the level of eHSP90 secretion. Significance was determined by the X^2^ test (*, *p* < 0.05; **, *p* < 0.005; ***, *p* < 0.001), comparing each variant to both wild type and p.C2579G (Hectd1*). See Table 3 for X^2^ and p values. Without ALLN treatment, the ability of the p.P2474L and p.T801I variants to suppress eHSP90 secretion was not significantly different from wild type HECTD1; the remaining variants showed a significant activity loss. Upon treatment with ALLN, all variants except the benign p.P2474L showed a considerable activity loss compared to the wild type

Our previous studies demonstrate that increased secretion of extracellular HSP90 (eHSP90) through the exosome pathway in *Hectd1* mutant cells stimulates abnormal Our previous studies demonstrate that increased secretion of extracellular HSP90 (eHSP90) through the exosome pathway in *Hectd1* mutant cells stimulates abnormal migration of the cranial mesenchyme in an explant assay (Sarkar and Zohn 2012). We showed that overexpression of HECTD1 prevents eHSP90 secretion in HEK293T cells and in response to stress induced by treating cells with the protease inhibitor ALLN (Sarkar and Zohn 2012). Thus, this assay can functionally test whether NTD-associated *HECTD1* missense variants retain the functionality of preventing eHSP90 secretion, a role related to NTDs. HEK293T cells were transfected with *Myc-Hsp90*, *CD63-GFP*, and the *HA-Hectd1* variant constructs. CD63-GFP is a well-characterized maker of exosomes secreted from the cells and was included to identify transfected cells. Immunostaining was done without permeabilization with detergent to detect only extracellular Myc-HSP90. Multiple independent reviewers scored the assay for the presence and intensity of eHSP90 staining on the surface of CD63-expressing cells. As shown in representative images in Fig. 4A, data were scored as “No secretion,” “Moderate secretion,” or “Excessive secretion.” Variants p.M392V, p.I906V, and p.P1835L demonstrate activity loss indicated by increased frequency of cells scored with “moderate” or “excessive” secretion of eHSP90 (Fig. 4B, No ALLN). This experiment was repeated in the presence of the protease inhibitor ALLN, which stimulates the secretion of eHsp90 (Sarkar and Zohn 2012). Upon the addition of ALLN, all NTD-associated sequence variants, but not the benign p.P2474L variant, showed reduced activity as measured by an increased proportion of cells scored with “moderate” or “excessive” secretion of eHSP90 compared to the wild type HECTD1.

## Discussion

This study identified five case-specific rare missense variants in the *HECTD1* gene in a well-described Chinese NTD cohort (Chen et al. 2018a; Chen et al. 2018b; Lei et al. 2019; Qiao et al. 2016; Shi et al. 2018; Ye et al. 2020). Our previous work demonstrated that *HECTD1* encodes an E3 Ubiquitin ligase required for neural tube closure in mouse models (Zohn et al. 2007). We now show evidence that *HECTD1* is a highly conserved gene across the animal kingdom. Moreover, the NTD-associated sequence variants identified in our study alter evolutionarily conserved amino acids within or adjacent to conserved functional domains of the protein. The missense variants identified in *HECTD1* in NTD cases are extremely rare in reference population databases, further supporting the possibility of pathogenicity. To generate experimental evidence supporting pathogenicity, NTD-associated *HECTD1* missense variants were expressed in HEK293T cells to determine if they altered protein expression or reduced gene function. Variant p.A1084T was expressed at significantly lower levels when transfected into HEK293T cells. All NTD-associated variants tested showed reduced activity in suppressing the secretion of eHSP90, a pathway regulated by HECTD1 previously linked to causing NTDs in our mouse model (Sarkar and Zohn 2012). Together, these data support a role for sequence variation in *HECTD1* contributing to NTDs in humans.

We originally identified *Hectd1* in a forward genetic screen in mice seeking new candidate genes for NTDs (Kasarskis et al. 1998; Zohn et al. 2005, 2007). Further study of our *Hectd1* mutant mouse lines found that NTDs result not from defects in the neural tissue but from disruption of cranial mesenchyme morphogenesis required for cranial neural fold elevation (Sarkar and Zohn 2012; Zohn et al. 2007). Mutation of *Hectd1* causes increased secretion of extracellular HSP90, resulting in altered morphogenesis of the cranial mesenchyme (Sarkar and Zohn 2012). The cranial mesenchyme lies underneath the cranial neural folds, and expansion of the cranial mesenchyme is believed to be required for the initial elevation of the cranial neural folds (Zohn and Sarkar 2012). Thus, in *Hectd1* mutant mouse embryos, increased eHSP90 secretion results in abnormal movement of the cranial mesenchyme, disrupting neural fold elevation and leading to NTDs. In the present study, the ability of HECTD1 to suppress eHSP90 secretion was utilized as an assay to determine if NTD-associated sequence variants resulted in loss of HECTD1 activity. All five variants identified reduced the activity of HECTD1 to prevent eHSP90 secretion, demonstrating that the sequence changes reduce activity related to the etiology of NTDs.

The *HECTD1* sequence variants identified in this study were found in the heterozygous state in NTD cases. While studies of our mutant mouse line causing NTDs utilized a homozygous model, mutation of one copy of *Hectd1* can result in NTDs in our model (Sarkar and Zohn 2012; Zohn et al. 2007). Approximately 5% of heterozygous *Hectd1*^*opm*^ mutant embryos exhibit exencephaly with a stop gain mutation at amino acid 144 (L144X) and 20% of heterozygous embryos with disruption of the HECT domain in the *Hectd1*^*Gt(XC266)Byg*^ line show NTDs. Since mutation of the HECT domain of HECT E3 ligases creates a dominant-negative protein by preserving the ligase’s interaction with substrates but preventing ubiquitination and degradation of substrates (Huibregtse et al. 1995; Treier et al. 1992), the *Hectd1*^*Gt(XC266)Byg*^ mutation likely has some dominant-negative activity.

Analysis of the gnomAD population database further supports the idea that sequence variation in *HECTD1* could cause disease in heterozygotes. We found that *HECTD1* has a high pLI score and a low LOEUF score and is intolerant to missense variation consistent with a haploinsufficient disease gene (Gudmundsson et al. 2022; Lek et al. 2016). One limitation of our study is the lack of available trio sequence data to determine the inheritance pattern or if the sequence variants occur de novo. Notably, the ethnically matched control cohort analysis did not identify the variants, which are extremely rare in the Asian population.

Genetic analysis of NTDs indicates oligogenic inheritance with environmental influences (Caiaffa et al. 2023; Chen et al. 2018c; Copp and Greene 2010; Ishida et al. 2018). HECTD1 is a ubiquitin ligase that regulates many pathways required for neural tube closure by targeting proteins for degradation or altering their function. Thus, it is also possible that sequence variation in *HECTD1* would interact with additional variants in these pathways to disrupt neural tube closure or contribute to human disease. HECTD1 plays a role in several signal transduction pathways essential for embryonic and fetal development, including Notch, Wnt, Retinoic acid, and estrogen receptor signaling (Chen and Greenwald 2014; Li et al. 2015; Oikonomaki et al. 2017; Sugrue et al. 2019; Tran et al. 2013). HECTD1 could broadly influence development by regulating the STRIPAK complex, chromatin remodeling, cholesterol export, and protein translation (Aleidi et al. 2015; Aleidi et al. 2018; Beard et al. 2016; Lampersberger et al. 2023; Li et al. 2015; Lu et al. 2022; Lv et al. 2021). HECTD1 influences proliferation, apoptosis, autophagy, DNA damage repair, and mitochondrial function (Beard et al. 2016; Bennett et al. 2020; Duhamel et al. 2018; Liao et al. 2023; Lu et al. 2022; Segref et al. 2014; Uemoto et al. 2022; Vaughan et al. 2022). HECTD1 is also involved in inflammation in astrocytes, microglia, and other cell types (Li et al. 2020; Liao et al. 2023; Tang et al. 2021; Zhang et al. 2020) and can regulate synaptic function by regulating the expression of glutamate transporter 1 (GLT-1) in astrocytes (Zeng et al. 2022). Intriguingly, the Chinese NTD cohort utilized in this study was collected in a region where folate deficiency is well documented (Liu et al. 2016; Meng et al. 2015). It would be intriguing to investigate whether the NTD-associated variants identified in our study reduce the activities of other HECTD1-regulated pathways. Additionally, we could explore if *HECTD1* variants interact with genetic variation or folate deficiency to cause NTDs.

In conclusion, we detected five case-specific rare missense variants in the *HECTD1* gene in a Chinese NTD cohort. All were functionally validated in assays demonstrating reduced protein expression of one variant and reduced function of all variants in an eHSP90 secretion assay linked to the mechanism of NTD formation in mouse models. Our results provide the first report of sequence variation in the *HECTD1* gene potentially contributing to human disease and the etiology of human NTDs.

## Figures and Tables

**Table 1.** *HECTD1* variants tested in this study.

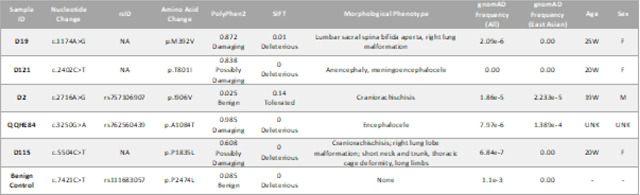

**Table 2.** Blastp results of HECTD1 against different species for comparative analyses in Figure 1B.

Species (common name)	Accession	Query Coverage	E-Value	Percent Identity	Total Score
**Mus musculus (mouse)**	NP_659037.2	100%	0	93.93%	5356
**Gallus gallus (Chicken)**	XP_421227.4	100%	0	95.60%	5177
**Xenopus laevis (Frog)**	XP_018087672.1	96%	0	89.55%	4554
**Danio rerio (Zebrafish)**	NP_001002504.2	100%	0	86.88%	4462
**Drosophila melanogaster (Fruit Fly)**	NP_609369.1	89%	0	53.50%	2481
**Monodelphis domestica (Opossum)**	XP_001364091.1	100%	0	97.82%	5301
**Lepisosteos oculatus (Spotted Gar)**	XP_015205628.1	100%	0	89.90%	4581
**Petromyzon marinus (Sea Lamprey)**	XP_032812070.l	96%	0	77.28%	3923
**Aplysia californica (Sea Slug)**	XP_005090788.1	86%	0	62.66%	2843
**Nematostella vectensis (Sea anemone)**	XP_048585862.1	84%	0	65.77%	2823
**Amphimedon queenslandica (Sponge)**	XP_019857021.1	74%	0	47.81%	1703
**Caenorhabditis elegans (Roundworm)**	NP_001368365.1	79%	0	52.31%	1788

**Table 3:** Summary of functional analysis shown in Fig. 4 to determine the effect of human NTD-associated *HECTD1* sequence variants on eHSP90 secretion. At baseline (No ALLN) or after treatment with ALLN when expressed in HEK293T cells

	No ALLN				ALLN treated			
	Compared to Hectd1 (WT)		Compared to Hectd1* (C2579G)		Compared to Hectd1 (WT)		Compared to Hectd1* (C2579G)	
	X^2^	P value	X^2^	P value	X^2^	P value	X^2^	P value
**Vector**	4.98	0.083	0.658	0.720	10	0.007	2.33	0.312
**Wildtype**	--	--	3.64	0.162	--	--	20	0
**C2579G**	3.64	0.162	--	--	20	0	--	--
**M392V**	6.60	0.037	3.13	0.209	17.2	0	1.18	0.554
**T801I**	0.834	0.66	1.88	0.392	14.7	0.001	5.19	0.075
**I906V**	12.7	0.002	6.49	0.039	11.2	0.004	1.79	0.408
**A1084T**	3.15	0.207	0	1	20.7	0	1.39	0.389
**P1835L**	11.3	0.004	3.32	0.190	12.5	0.002	4.48	0.107
**P2474L**	2.83	0.243	3.71	0.157	5.26	0.072	9.35	0.009

## Data Availability

The datasets generated during and/or analyzed in this study are available from the corresponding author upon reasonable request.

## References

[1] Adzhubei IA, Schmidt S, Peshkin L, Ramensky VE, Gerasimova A, Bork P, Kondrashov AS, Sunyaev SR (2010) A method and server for predicting damaging missense mutations. Nat Methods 7: 248–9. doi: 10.1038/nmeth0410-248 20354512 PMC2855889

[2] Agarwal I, Fuller ZL, Myers SR, Przeworski M (2023) Relating pathogenic loss-of-function mutations in humans to their evolutionary fitness costs. Elife 12. doi: 10.7554/eLife.83172 PMC9937649 36648429

[3] Aleidi SM, Howe V, Sharpe LJ, Yang A, Rao G, Brown AJ, Gelissen IC (2015) The E3 ubiquitin ligases, HUWE1 and NEDD4–1, are involved in the post-translational regulation of the ABCG1 and ABCG4 lipid transporters. J Biol Chem 290: 24604–13. doi: 10.1074/jbc.M115.675579 26296893 PMC4591838

[4] Aleidi SM, Yang A, Sharpe LJ, Rao G, Cochran BJ, Rye KA, Kockx M, Brown AJ, Gelissen IC (2018) The E3 ubiquitin ligase, HECTD1, is involved in ABCA1-mediated cholesterol export from macrophages. Biochim Biophys Acta Mol Cell Biol Lipids 1863: 359–368. doi: 10.1016/j.bbalip.2017.12.011 29306077

[5] Bartsch O, Kirmes I, Thiede A, Lechno S, Gocan H, Florian IS, Haaf T, Zechner U, Sabova L, Horn F (2012) Novel VANGL1 Gene Mutations in 144 Slovakian, Romanian and German Patients with Neural Tube Defects. Mol Syndromol 3: 76–81. doi: 10.1159/000339668 23326252 PMC3542939

[6] Beard SM, Smit RB, Chan BG, Mains PE (2016) Regulation of the MEI-1/MEI-2 Microtubule-Severing Katanin Complex in Early Caenorhabditis elegans Development. G3 (Bethesda) 6: 3257–3268. doi: 10.1534/g3.116.031666 27527792 PMC5068946

[7] Bennett L, Madders E, Parsons JL (2020) HECTD1 promotes base excision repair in nucleosomes through chromatin remodelling. Nucleic Acids Res 48: 1301–1313. doi: 10.1093/nar/gkz1129 31799632 PMC7026656

[8] Cai C, Shi O, Wang B, Chang B, Yang R, Wang Y, Wang F, Shen C (2014) Association between VANGL1 gene polymorphisms and neural tube defects. Neuropediatrics 45: 234–9. doi: 10.1055/s-0033-1364103 24407469

[9] Caiaffa CD, Fonteles CSR, Yunping L, Finnell RH (2023) Gene-environment interactions underlying the etiology of neural tube defects. Curr Top Dev Biol 152: 193–220. doi: 10.1016/bs.ctdb.2022.10.007 36707212 PMC12619917

[10] Chen S, Francioli LC, Goodrich JK, Collins RL, Kanai M, Wang Q, Alföldi J, Watts NA, Vittal C, Gauthier LD, Poterba T, Wilson MW, Tarasova Y, Phu W, Yohannes MT, Koenig Z, Farjoun Y, Banks E, Donnelly S, Gabriel S, Gupta N, Ferriera S, Tolonen C, Novod S, Bergelson L, Roazen D, Ruano-Rubio V, Covarrubias M, Llanwarne C, Petrillo N, Wade G, Jeandet T, Munshi R, Tibbetts K, Consortium gP, O’Donnell-Luria A, Solomonson M, Seed C, Martin AR, Talkowski ME, Rehm HL, Daly MJ, Tiao G, Neale BM, MacArthur DG, Karczewski KJ (2022) A genome-wide mutational constraint map quantified from variation in 76,156 human genomes. bioRxiv: 2022.03.20.485034. doi: 10.1101/2022.03.20.485034

[11] Chen Y, Greenwald I (2014) hecd-1 modulates notch activity in Caenorhabditis elegans. G3 (Bethesda) 5: 353–9. doi: 10.1534/g3.114.015321 25552605 PMC4349089

[12] Chen Z, Kuang L, Finnell RH, Wang H (2018a) Genetic and functional analysis of SHROOM1–4 in a Chinese neural tube defect cohort. Hum Genet 137: 195–202. doi: 10.1007/s00439-017-1864-x 29423651 PMC5876139

[13] Chen Z, Lei Y, Cao X, Zheng Y, Wang F, Bao Y, Peng R, Finnell RH, Zhang T, Wang H (2018b) Genetic analysis of Wnt/PCP genes in neural tube defects. BMC Med Genomics 11: 38. doi: 10.1186/s12920-018-0355-9 29618362 PMC5885375

[14] Chen Z, Lei Y, Zheng Y, Aguiar-Pulido V, Ross ME, Peng R, Jin L, Zhang T, Finnell RH, Wang H (2018c) Threshold for neural tube defect risk by accumulated singleton loss-of-function variants. Cell Res 28: 1039–1041. doi: 10.1038/s41422-018-0061-3 29976953 PMC6170406

[15] Cheng C, Zhao S, Zhu X, Yang F, Wang W, Feng Q, Liu Y, Huang H, Chen X (2021) The VANGL1 P384R variant cause both neural tube defect and Klippel-Feil syndrome. Mol Genet Genomic Med 9: e1710. doi: 10.1002/mgg3.1710 34014041 PMC8372072

[16] Copp AJ, Adzick NS, Chitty LS, Fletcher JM, Holmbeck GN, Shaw GM (2015) Spina bifida. Nat Rev Dis Primers 1: 15007. doi: 10.1038/nrdp.2015.7 27189655 PMC4898641

[17] Copp AJ, Greene ND (2010) Genetics and development of neural tube defects. J Pathol 220: 217–30. doi: 10.1002/path.2643 19918803 PMC4239538

[18] De Marco P, Merello E, Piatelli G, Cama A, Kibar Z, Capra V (2014) Planar cell polarity gene mutations contribute to the etiology of human neural tube defects in our population. Birth Defects Res A Clin Mol Teratol 100: 633–41. doi: 10.1002/bdra.23255 24838524

[19] Dilworth D, Upadhyay SK, Bonnafous P, Edoo AB, Bourbigot S, Pesek-Jardim F, Gudavicius G, Serpa JJ, Petrotchenko EV, Borchers CH, Nelson CJ, Mackereth CD (2017) The basic tilted helix bundle domain of the prolyl isomerase FKBP25 is a novel double-stranded RNA binding module. Nucleic Acids Res 45: 11989–12004. doi: 10.1093/nar/gkx852 29036638 PMC5714180

[20] Doudney K, Ybot-Gonzalez P, Paternotte C, Stevenson RE, Greene ND, Moore GE, Copp AJ, Stanier P (2005) Analysis of the planar cell polarity gene Vangl2 and its co-expressed paralogue Vangl1 in neural tube defect patients. Am J Med Genet A 136: 90–2. doi: 10.1002/ajmg.a.30766 15952208

[21] Duhamel S, Goyette MA, Thibault MP, Filion D, Gaboury L, Cote JF (2018) The E3 Ubiquitin Ligase HectD1 Suppresses EMT and Metastasis by Targeting the +TIP ACF7 for Degradation. Cell Rep 22: 1016–1030. doi: 10.1016/j.celrep.2017.12.096 29386124

[22] Fatima U, Khan S, Riaz SU, Mehdi H, Iftikhar M, Fatima N (2022) Myelomeningocele among Pakistani population. J Pak Med Assoc 72: 874–877. doi: 10.47391/JPMA.04-611 35713047

[23] Greene ND, Copp AJ (2014) Neural tube defects. Annu Rev Neurosci 37: 221–42. doi: 10.1146/annurev-neuro-062012-170354 25032496 PMC4486472

[24] Gudmundsson S, Singer-Berk M, Watts NA, Phu W, Goodrich JK, Solomonson M, Genome Aggregation Database C, Rehm HL, MacArthur DG, O’Donnell-Luria A (2022) Variant interpretation using population databases: Lessons from gnomAD. Hum Mutat 43: 1012–1030. doi: 10.1002/humu.24309 34859531 PMC9160216

[25] Harris MJ, Juriloff DM (2007) Mouse mutants with neural tube closure defects and their role in understanding human neural tube defects. Birth Defects Res A Clin Mol Teratol 79: 187–210. doi: 10.1002/bdra.20333 17177317

[26] Harris MJ, Juriloff DM (2010) An update to the list of mouse mutants with neural tube closure defects and advances toward a complete genetic perspective of neural tube closure. Birth Defects Res A Clin Mol Teratol 88: 653–69. doi: 10.1002/bdra.20676 20740593

[27] Huibregtse JM, Scheffner M, Beaudenon S, Howley PM (1995) A family of proteins structurally and functionally related to the E6-AP ubiquitin-protein ligase. Proc Natl Acad Sci U S A 92: 2563–7. doi: 10.1073/pnas.92.7.2563 7708685 PMC42258

[28] Humphries AC, Narang S, Mlodzik M (2020) Mutations associated with human neural tube defects display disrupted planar cell polarity in Drosophila. Elife 9. doi: 10.7554/eLife.53532 PMC7180057 32234212

[29] Iliescu A, Gravel M, Horth C, Gros P (2014) Independent mutations at Arg181 and Arg274 of Vangl proteins that are associated with neural tube defects in humans decrease protein stability and impair membrane targeting. Biochemistry 53: 5356–64. doi: 10.1021/bi500400g 25068569

[30] Iliescu A, Gravel M, Horth C, Kibar Z, Gros P (2011) Loss of membrane targeting of Vangl proteins causes neural tube defects. Biochemistry 50: 795–804. doi: 10.1021/bi101286d 21142127

[31] Ishida M, Cullup T, Boustred C, James C, Docker J, English C, Gosgene, Lench N, Copp AJ, Moore GE, Greene NDE, Stanier P (2018) A targeted sequencing panel identifies rare damaging variants in multiple genes in the cranial neural tube defect, anencephaly. Clin Genet 93: 870–879. doi: 10.1111/cge.13189 29205322 PMC5887939

[32] Jorde LB, Fineman RM, Martin RA (1983) Epidemiology and genetics of neural tube defects: an application of the Utah Genealogical Data Base. Am J Phys Anthropol 62: 23–31. doi: 10.1002/ajpa.1330620106 6353932

[33] Juriloff DM, Harris MJ (2012) A consideration of the evidence that genetic defects in planar cell polarity contribute to the etiology of human neural tube defects. Birth Defects Res A Clin Mol Teratol 94: 824–40. doi: 10.1002/bdra.23079 23024041

[34] Karczewski KJ, Francioli LC, Tiao G, Cummings BB, Alfoldi J, Wang Q, Collins RL, Laricchia KM, Ganna A, Birnbaum DP, Gauthier LD, Brand H, Solomonson M, Watts NA, Rhodes D, Singer-Berk M, England EM, Seaby EG, Kosmicki JA, Walters RK, Tashman K, Farjoun Y, Banks E, Poterba T, Wang A, Seed C, Whiffin N, Chong JX, Samocha KE, Pierce-Hoffman E, Zappala Z, O’Donnell-Luria AH, Minikel EV, Weisburd B, Lek M, Ware JS, Vittal C, Armean IM, Bergelson L, Cibulskis K, Connolly KM, Covarrubias M, Donnelly S, Ferriera S, Gabriel S, Gentry J, Gupta N, Jeandet T, Kaplan D, Llanwarne C, Munshi R, Novod S, Petrillo N, Roazen D, Ruano-Rubio V, Saltzman A, Schleicher M, Soto J, Tibbetts K, Tolonen C, Wade G, Talkowski ME, Genome Aggregation Database C, Neale BM, Daly MJ, MacArthur DG (2020) The mutational constraint spectrum quantified from variation in 141,456 humans. Nature 581: 434–443. doi: 10.1038/s41586-020-2308-7 32461654 PMC7334197

[35] Kasarskis A, Manova K, Anderson KV (1998) A phenotype-based screen for embryonic lethal mutations in the mouse. Proc Natl Acad Sci U S A 95: 7485–90. doi: 10.1073/pnas.95.13.7485 9636176 PMC22659

[36] Kibar Z, Bosoi CM, Kooistra M, Salem S, Finnell RH, De Marco P, Merello E, Bassuk AG, Capra V, Gros P (2009) Novel mutations in VANGL1 in neural tube defects. Hum Mutat 30: E706–15. doi: 10.1002/humu.21026 19319979 PMC2885434

[37] Kibar Z, Torban E, McDearmid JR, Reynolds A, Berghout J, Mathieu M, Kirillova I, De Marco P, Merello E, Hayes JM, Wallingford JB, Drapeau P, Capra V, Gros P (2007) Mutations in VANGL1 associated with neural-tube defects. N Engl J Med 356: 1432–7. doi: 10.1056/NEJMoa060651 17409324

[38] Lampersberger L, Conte F, Ghosh S, Xiao Y, Price J, Jordan D, Matus DQ, Sarkies P, Beli P, Miska EA, Burton NO (2023) Loss of the E3 ubiquitin ligases UBR-5 or HECD-1 restores Caenorhabditis elegans development in the absence of SWI/SNF function. Proc Natl Acad Sci U S A 120: e2217992120. doi: 10.1073/pnas.2217992120 36689659 PMC9945973

[39] Lei Y, Kim SE, Chen Z, Cao X, Zhu H, Yang W, Shaw GM, Zheng Y, Zhang T, Wang HY, Finnell RH (2019) Variants identified in PTK7 associated with neural tube defects. Mol Genet Genomic Med 7: e00584. doi: 10.1002/mgg3.584 30689296 PMC6465732

[40] Lek M, Karczewski KJ, Minikel EV, Samocha KE, Banks E, Fennell T, O’Donnell-Luria AH, Ware JS, Hill AJ, Cummings BB, Tukiainen T, Birnbaum DP, Kosmicki JA, Duncan LE, Estrada K, Zhao F, Zou J, Pierce-Hoffman E, Berghout J, Cooper DN, Deflaux N, DePristo M, Do R, Flannick J, Fromer M, Gauthier L, Goldstein J, Gupta N, Howrigan D, Kiezun A, Kurki MI, Moonshine AL, Natarajan P, Orozco L, Peloso GM, Poplin R, Rivas MA, Ruano-Rubio V, Rose SA, Ruderfer DM, Shakir K, Stenson PD, Stevens C, Thomas BP, Tiao G, Tusie-Luna MT, Weisburd B, Won HH, Yu D, Altshuler DM, Ardissino D, Boehnke M, Danesh J, Donnelly S, Elosua R, Florez JC, Gabriel SB, Getz G, Glatt SJ, Hultman CM, Kathiresan S, Laakso M, McCarroll S, McCarthy MI, McGovern D, McPherson R, Neale BM, Palotie A, Purcell SM, Saleheen D, Scharf JM, Sklar P, Sullivan PF, Tuomilehto J, Tsuang MT, Watkins HC, Wilson JG, Daly MJ, MacArthur DG, Exome Aggregation C (2016) Analysis of protein-coding genetic variation in 60,706 humans. Nature 536: 285–91. doi: 10.1038/nature19057 27535533 PMC5018207

[41] Li W, Hu Y, Oh S, Ma Q, Merkurjev D, Song X, Zhou X, Liu Z, Tanasa B, He X, Chen AY, Ohgi K, Zhang J, Liu W, Rosenfeld MG (2015) Condensin I and II Complexes License Full Estrogen Receptor alpha-Dependent Enhancer Activation. Mol Cell 59: 188–202. doi: 10.1016/j.molcel.2015.06.002 26166704 PMC5770188

[42] Li Y, Huang B, Yang H, Kan S, Yao Y, Liu X, Pu S, He G, Khan TM, Qi G, Zhou Z, Shu W, Chen M (2020) Latexin deficiency in mice up-regulates inflammation and aggravates colitis through HECTD1/Rps3/NF-kappaB pathway. Sci Rep 10: 9868. doi: 10.1038/s41598-020-66789-x 32555320 PMC7299958

[43] Li Z, Ren A, Zhang L, Ye R, Li S, Zheng J, Hong S, Wang T, Li Z (2006) Extremely high prevalence of neural tube defects in a 4-county area in Shanxi Province, China. Birth Defects Res A Clin Mol Teratol 76: 237–40. doi: 10.1002/bdra.20248 16575897

[44] Liao S, Zheng Q, Shen H, Yang G, Xu Y, Zhang X, Ouyang H, Pan Z (2023) HECTD1-Mediated Ubiquitination and Degradation of Rubicon Regulates Autophagy and Osteoarthritis Pathogenesis. Arthritis Rheumatol 75: 387–400. doi: 10.1002/art.42369 36121967

[45] Liu J, Zhang L, Li Z, Jin L, Zhang Y, Ye R, Liu J, Ren A (2016) Prevalence and trend of neural tube defects in five counties in Shanxi province of Northern China, 2000 to 2014. Birth Defects Res A Clin Mol Teratol 106: 267–74. doi: 10.1002/bdra.23486 26879384

[46] Lu T, Smit RB, Soueid H, Mains PE (2022) STRIPAK regulation of katanin microtubule severing in the Caenorhabditis elegans embryo. Genetics 221. doi: 10.1093/genetics/iyac043 PMC9071564 35298637

[47] Lupo PJ, Agopian AJ, Castillo H, Castillo J, Clayton GH, Dosa NP, Hopson B, Joseph DB, Rocque BG, Walker WO, Wiener JS, Mitchell LE (2017) Genetic epidemiology of neural tube defects. J Pediatr Rehabil Med 10: 189–194. doi: 10.3233/PRM-170456 29125517 PMC8085973

[48] Lv K, Gong C, Antony C, Han X, Ren JG, Donaghy R, Cheng Y, Pellegrino S, Warren AJ, Paralkar VR, Tong W (2021) HectD1 controls hematopoietic stem cell regeneration by coordinating ribosome assembly and protein synthesis. Cell Stem Cell 28: 1275–1290 e9. doi: 10.1016/j.stem.2021.02.008 33711283 PMC8254759

[49] Meng Q, Zhang L, Liu J, Li Z, Jin L, Zhang Y, Wang L, Ren A (2015) Dietary folate intake levels in rural women immediately before pregnancy in Northern China. Birth Defects Res A Clin Mol Teratol 103: 27–36. doi: 10.1002/bdra.23280 25066482

[50] Merello E, Mascelli S, Raso A, Piatelli G, Consales A, Cama A, Kibar Z, Capra V, Marco PD (2015) Expanding the mutational spectrum associated to neural tube defects: literature revision and description of novel VANGL1 mutations. Birth Defects Res A Clin Mol Teratol 103: 51–61. doi: 10.1002/bdra.23305 25208524

[51] Morris JK, Wald NJ (2007) Prevalence of neural tube defect pregnancies in England and Wales from 1964 to 2004. J Med Screen 14: 55–9. doi: 10.1258/096914107781261945 17626701

[52] Morris-Wiman J, Brinkley LL (1990a) Changes in mesenchymal cell and hyaluronate distribution correlate with in vivo elevation of the mouse mesencephalic neural folds. Anat Rec 226: 383–95. 2327607 10.1002/ar.1092260316

[53] Morris-Wiman J, Brinkley LL (1990b) The role of the mesenchyme in mouse neural fold elevation. I. Patterns of mesenchymal cell distribution and proliferation in embryos developing in vitro. Am J Anat 188: 121–32. 2375278 10.1002/aja.1001880203

[54] Morris-Wiman J, Brinkley LL (1990c) The role of the mesenchyme in mouse neural fold elevation. II. Patterns of hyaluronate synthesis and distribution in embryos developing in vitro. Am J Anat 188: 133–47. 2375279 10.1002/aja.1001880204

[55] Morriss GM, Solursh M (1978a) Regional differences in mesenchymal cell morphology and glycosaminoglycans in early neural-fold stage rat embryos. J Embryol Exp Morphol 46: 37–52. 702034

[56] Morriss GM, Solursh M (1978b) The role of primary mesenchyme in normal and abnormal morphogenesis of mammalian neural folds. Zoon 6: 33–38.

[57] Ng PC, Henikoff S (2003) SIFT: Predicting amino acid changes that affect protein function. Nucleic Acids Res 31: 3812–4. doi: 10.1093/nar/gkg509 12824425 PMC168916

[58] Oikonomaki M, Bady P, Hegi ME (2017) Ubiquitin Specific Peptidase 15 (USP15) suppresses glioblastoma cell growth via stabilization of HECTD1 E3 ligase attenuating WNT pathway activity. Oncotarget 8: 110490–110502. doi: 10.18632/oncotarget.22798 29299163 PMC5746398

[59] Petrovski S, Wang Q, Heinzen EL, Allen AS, Goldstein DB (2013) Genic intolerance to functional variation and the interpretation of personal genomes. PLoS Genet 9: e1003709. doi: 10.1371/journal.pgen.1003709 23990802 PMC3749936

[60] Qiao X, Liu Y, Li P, Chen Z, Li H, Yang X, Finnell RH, Yang Z, Zhang T, Qiao B, Zheng Y, Wang H (2016) Genetic analysis of rare coding mutations of CELSR1–3 in congenital heart and neural tube defects in Chinese people. Clin Sci (Lond) 130: 2329–2340. doi: 10.1042/CS20160686 27756857

[61] Reynolds A, McDearmid JR, Lachance S, De Marco P, Merello E, Capra V, Gros P, Drapeau P, Kibar Z (2010) VANGL1 rare variants associated with neural tube defects affect convergent extension in zebrafish. Mech Dev 127: 385–92. doi: 10.1016/j.mod.2009.12.002 20043994 PMC2965831

[62] Sarkar AA, Zohn IE (2012) Hectd1 regulates intracellular localization and secretion of Hsp90 to control cellular behavior of the cranial mesenchyme. J Cell Biol 196: 789–800. doi: 10.1083/jcb.201105101 22431752 PMC3308699

[63] Segref A, Kevei E, Pokrzywa W, Schmeisser K, Mansfeld J, Livnat-Levanon N, Ensenauer R, Glickman MH, Ristow M, Hoppe T (2014) Pathogenesis of human mitochondrial diseases is modulated by reduced activity of the ubiquitin/proteasome system. Cell Metab 19: 642–52. doi: 10.1016/j.cmet.2014.01.016 24703696

[64] Shi Z, Yang X, Li BB, Chen S, Yang L, Cheng L, Zhang T, Wang H, Zheng Y (2018) Novel Mutation of LRP6 Identified in Chinese Han Population Links Canonical WNT Signaling to Neural Tube Defects. Birth Defects Res 110: 63–71. doi: 10.1002/bdr2.1122 28960852

[65] Starr DA, Fridolfsson HN (2010) Interactions between nuclei and the cytoskeleton are mediated by SUN-KASH nuclear-envelope bridges. Annu Rev Cell Dev Biol 26: 421–44. doi: 10.1146/annurev-cellbio-100109-104037 20507227 PMC4053175

[66] Stefely JA, Zhang Y, Freiberger EC, Kwiecien NW, Thomas HE, Davis AM, Lowry ND, Vincent CE, Shishkova E, Clark NA, Medvedovic M, Coon JJ, Pagliarini DJ, Mercer CA (2020) Mass spectrometry proteomics reveals a function for mammalian CALCOCO1 in MTOR-regulated selective autophagy. Autophagy 16: 2219–2237. doi: 10.1080/15548627.2020.1719746 31971854 PMC7751563

[67] Sugrue KF, Sarkar AA, Leatherbury L, Zohn IE (2019) The ubiquitin ligase HECTD1 promotes retinoic acid signaling required for development of the aortic arch. Dis Model Mech 12. doi: 10.1242/dmm.036491 PMC6361158 30578278

[68] Tang Y, Zhou M, Huang R, Shen L, Yang L, Zhou Z, Ren H, Bai Y (2021) Involvement of HECTD1 in LPS-induced astrocyte activation via sigma-1R-JNK/p38-FOXJ2 axis. Cell Biosci 11: 62. doi: 10.1186/s13578-021-00572-x 33781347 PMC8008527

[69] Tian T, Lei Y, Chen Y, Guo Y, Jin L, Finnell RH, Wang L, Ren A (2020a) Rare copy number variations of planar cell polarity genes are associated with human neural tube defects. Neurogenetics 21: 217–225. doi: 10.1007/s10048-020-00613-6 32388773

[70] Tian T, Lei Y, Chen Y, Karki M, Jin L, Finnell RH, Wang L, Ren A (2020b) Somatic mutations in planar cell polarity genes in neural tissue from human fetuses with neural tube defects. Hum Genet 139: 1299–1314. doi: 10.1007/s00439-020-02172-0 32356230 PMC7487040

[71] Torban E, Patenaude AM, Leclerc S, Rakowiecki S, Gauthier S, Andelfinger G, Epstein DJ, Gros P (2008) Genetic interaction between members of the Vangl family causes neural tube defects in mice. Proc Natl Acad Sci U S A 105: 3449–54. doi: 10.1073/pnas.0712126105 18296642 PMC2265143

[72] Toriello HV, Higgins JV (1983) Occurrence of neural tube defects among first-, second-, and third- degree relatives of probands: results of a United States study. Am J Med Genet 15: 601–6. doi: 10.1002/ajmg.1320150409 6614048

[73] Tran H, Bustos D, Yeh R, Rubinfeld B, Lam C, Shriver S, Zilberleyb I, Lee MW, Phu L, Sarkar AA, Zohn IE, Wertz IE, Kirkpatrick DS, Polakis P (2013) HectD1 E3 ligase modifies adenomatous polyposis coli (APC) with polyubiquitin to promote the APC-axin interaction. J Biol Chem 288: 3753–67. doi: 10.1074/jbc.M112.415240 23277359 PMC3567630

[74] Treier M, Seufert W, Jentsch S (1992) Drosophila UbcD1 encodes a highly conserved ubiquitin-conjugating enzyme involved in selective protein degradation. EMBO J 11: 367–72. doi: 10.1002/j.1460-2075.1992.tb05059.x 1310935 PMC556457

[75] Uemoto Y, Katsuta E, Kondo N, Wanifuchi-Endo Y, Fujita T, Asano T, Hisada T, Terada M, Kato A, Okuda K, Sugiura H, Komura M, Kato H, Osaga S, Takahashi S, Toyama T (2022) Low HECTD1 mRNA expression is associated with poor prognosis and may be correlated with increased mitochondrial respiratory function in breast cancer. Am J Cancer Res 12: 1593–1605. 35530276 PMC9077061

[76] Vaughan N, Scholz N, Lindon C, Licchesi JDF (2022) The E3 ubiquitin ligase HECTD1 contributes to cell proliferation through an effect on mitosis. Sci Rep 12: 13160. doi: 10.1038/s41598-022-16965-y 35915203 PMC9343455

[77] Wallingford JB, Niswander LA, Shaw GM, Finnell RH (2013) The continuing challenge of understanding, preventing, and treating neural tube defects. Science 339: 1222002. doi: 10.1126/science.1222002 23449594 PMC3677196

[78] Wang L, Xiao Y, Tian T, Jin L, Lei Y, Finnell RH, Ren A (2018) Digenic variants of planar cell polarity genes in human neural tube defect patients. Mol Genet Metab 124: 94–100. doi: 10.1016/j.ymgme.2018.03.005 29573971 PMC5966321

[79] Wilde JJ, Petersen JR, Niswander L (2014) Genetic, epigenetic, and environmental contributions to neural tube closure. Annu Rev Genet 48: 583–611. doi: 10.1146/annurev-genet-120213-092208 25292356 PMC4649936

[80] Williams J, Mai CT, Mulinare J, Isenburg J, Flood TJ, Ethen M, Frohnert B, Kirby RS, Centers for Disease C, Prevention (2015) Updated estimates of neural tube defects prevented by mandatory folic Acid fortification - United States, 1995–2011. MMWR Morb Mortal Wkly Rep 64: 1–5. 25590678 PMC4584791

[81] Wolujewicz P, Ross ME (2019) The search for genetic determinants of human neural tube defects. Curr Opin Pediatr 31: 739–746. doi: 10.1097/MOP.0000000000000817 31693581 PMC6993899

[82] Ye J, Tong Y, Lv J, Peng R, Chen S, Kuang L, Su K, Zheng Y, Zhang T, Zhang F, Jin L, Yang X, Wang H (2020) Rare mutations in the autophagy-regulating gene AMBRA1 contribute to human neural tube defects. Hum Mutat 41: 1383–1393. doi: 10.1002/humu.24028 32333458

[83] Zeng X, Dong X, Xiao Q, Yao J (2022) Vitamin C Inhibits Ubiquitination of Glutamate Transporter 1 (GLT-1) in Astrocytes by Downregulating HECTD1. ACS Chem Neurosci 13: 676–687. doi: 10.1021/acschemneuro.1c00845 35148069

[84] Zhang Y, Du L, Bai Y, Han B, He C, Gong L, Huang R, Shen L, Chao J, Liu P, Zhang H, Zhang H, Gu L, Li J, Hu G, Xie C, Zhang Z, Yao H (2020) CircDYM ameliorates depressive-like behavior by targeting miR-9 to regulate microglial activation via HSP90 ubiquitination. Mol Psychiatry 25: 1175–1190. doi: 10.1038/s41380-018-0285-0 30413800 PMC7244405

[85] Zohn IE (2012) Mouse as a model for multifactorial inheritance of neural tube defects. Birth Defects Res C Embryo Today 96: 193–205. doi: 10.1002/bdrc.21011 22692891

[86] Zohn IE, Anderson KV, Niswander L (2005) Using genomewide mutagenesis screens to identify the genes required for neural tube closure in the mouse. Birth Defects Res A Clin Mol Teratol 73: 583–90. doi: 10.1002/bdra.20164 15971254

[87] Zohn IE, Anderson KV, Niswander L (2007) The Hectd1 ubiquitin ligase is required for development of the head mesenchyme and neural tube closure. Dev Biol 306: 208–21. doi: 10.1016/j.ydbio.2007.03.018 17442300 PMC2730518

[88] Zohn IE, Sarkar AA (2008) Modeling neural tube defects in the mouse. Curr Top Dev Biol 84: 1–35. doi: 10.1016/S0070-2153(08)00601-7 19186242

[89] Zohn IE, Sarkar AA (2012) Does the cranial mesenchyme contribute to neural fold elevation during neurulation? Birth Defects Res A Clin Mol Teratol 94: 841–8. doi: 10.1002/bdra.23073 22945385 PMC3473154

